# Epstein–Barr virus reactivation influences clonal evolution in human herpesvirus‐8‐related lymphoproliferative disorders

**DOI:** 10.1111/his.14551

**Published:** 2021-10-04

**Authors:** Massimo Granai, Mattia Facchetti, Virginia Mancini, Jacqueline Goedhals, Alicia Sherriff, Lucia Mundo, Cristiana Bellan, Teresa Amato, Ester Sorrentino, Marco Ungari, Martine Raphael, Lorenzo Leoncini, Fabio Facchetti, Stefano Lazzi

**Affiliations:** ^1^ Section of Pathology Department of Medical Biotechnology University of Siena Siena Italy; ^2^ Institute of Pathology and Neuropathology University Hospital and Comprehensive Cancer Centre Tübingen Tübingen Germany; ^3^ Section of Pathology Department of Molecular and Translational Medicine University of Brescia Brescia Italy; ^4^ Department of Pathology Faculty of Health Sciences University of the Free State Bloemfontein South Africa; ^5^ Department of Oncology Faculty of Health Sciences University of the Free State Bloemfontein South Africa; ^6^ Health Research Institute University of Limerick Limerick Ireland; ^7^ Department of Pathology Cremona Hospital Cremona Italy; ^8^ University of Paris Sud Paris France

**Keywords:** Castleman’s disease, EBV, germinotropic lymphoproliferative disorder, KSHV/HHV8, lymphoma, pleural effusion

## Abstract

**Background:**

Human herpesvirus‐8 (HHV8) is a lymphotropic virus associated with different lymphoproliferative disorders, including primary effusion lymphoma (PEL), multicentric Castleman’s disease (MCD), diffuse large B‐cell lymphomas, not otherwise specified, and the rare entity known as germinotropic lymphoproliferative disorder (GLPD). In PELs and GLPD the neoplastic cells also contain Epstein–Barr virus (EBV). In addition, occasional cases with atypical and overlapping features among these entities have been recognised, suggesting that the spectrum of the HHV8‐related lesions may not be fully characterised.

**Aims:**

Here, we report two cases of lymphoproliferative disorder associated with HHV8 and EBV that further expand the spectrum of HHV8/EBV‐positive lymphoproliferative disease.

**Methods and results:**

Case 1 represented HHV8/EBV‐positive extracavitary nodal PEL followed by pleural PEL. The striking characteristic of this case was the almost focal and intrasinusoidal localisation of the neoplastic cells and the association with Castleman’s disease features. In the second case, we found the entire spectrum of HHV8‐related disorders, i.e. MCD, GLPD, and PEL, coexisting in the same lymph node, underlining the variability, possible overlap and evolution among these entities. Both cases were well analysed with immunohistochemistry, determination of the EBV latency programme, and molecular analysis for clonality of immnoglobulin genes. In both patients, the disease followed an unexpected indolent course, both being still alive after 8 and 12 months, respectively.

**Conclusion:**

Our findings represent further evidence of the overlap among HHV8/EBV‐positive lymphoproliferative disorders, and underline a grey zone that requires further study; they further confirm the experimental evidence that lytic EBV replication influences HHV8‐related tumorigenesis.

## Introduction

Since the discovery of human herpesvirus‐8 (HHV8) as the causative agent of Kaposi’s sarcoma in 1994,^1^ HHV8 has been identified in several different types of lymphoproliferative disorder (LPD), with distinctive clinicopathological features, including the propensity for Epstein–Barr virus (EBV) coinfection.^2^, ^3^, ^4^

In particular, HHV8 has been identified in multicentric Castleman’s disease (MCD),^5^ in the rare entity known as germinotropic LPD (GLPD),^6^ in primary effusion lymphoma (PEL) and its solid variant [extracavitary PEL (EC‐PEL)],^7^, ^8^ and in HHV8‐positive diffuse large B‐cell lymphoma, not otherwise specified.^9^ In particular, in PELs and GLPD the neoplastic cells also contain EBV, which itself causes 1–2% of the total human cancer burden worldwide.^6^, ^7^

Except for GLPD, the majority of these disorders arise in human immunodeficiency virus (HIV)‐positive patients,^10^, ^11^ but cases in HIV‐seronegative patients have also been reported.^12^, ^13^

Similarly to what occurs with EBV (human herpesvirus‐4),^14^ the oncogenic transformation of HHV8 occurs following the initial infection, when the virus establishes a latent phase. HHV8‐positive lymphoproliferations may originate from B cells in different stages of B‐cell differentiation.^15^ The maturation stage of HHV8‐positive B cells might thus determine the heterogeneity of HHV8‐related lymphoid diseases.^4^, ^15^ Latent and lytic proteins are detected in HHV8‐related neoplasia and LPDs, both contributing to neoplastic transformation in a paracrine or autocrine fashion.^16^ Unlike EBV‐related lymphomas, HHV8‐related neoplasms are not associated with different latency programmes.^16^

How HHV8 and EBV cooperate to promote tumorigenesis remains unclear, but recent experimental data suggest that EBV/HHV8 coinfection enhances HHV8 persistence and tumorigenesis.^17^, ^18^, ^19^, ^20^ In particular, EBV/Kaposi’s sarcoma‐associated herpesvirus coinfection in mice with reconstituted human immune system components has revealed a role for lytic EBV replication during virus‐associated lymphomagenesis, which might even be diagnostically useful for predicting the risk of malignancy development.^17^, ^18^, ^19^

In humans, occasional cases with atypical and overlapping features among HHV8/EBV‐related LPDs have been recognised, such as lesions intermediate between MCD and GLPD in HIV‐positive patients, or GLPD that progresses to high‐grade EBV or HHV8‐positive lymphoma.^21^, ^22^, ^23^, ^24^, ^25^, ^26^ These cases represent diagnostic challenges, and suggest that the spectrum of HHV8‐related lesions and the interaction with EBV infection may not be fully characterised.

Here, we report two cases that showed Castleman’s disease features in association with HHV8/EBV‐positive lymphoproliferations, characterised by early sinusal and germinal centre involvement of the lymph node by atypical cells with plasmablastic morphology. Both cases were analysed with morphology, immunohistochemistry, determination of the EBV latency programme, and molecular analysis for clonality of immunoglobulin genes. Our findings expand the clinical and pathological spectrum of HHV8‐related LPDS, underlying a grey zone and possible evolution among them.

## Materials and methods

One lymph node from each patient was excised and, after fixation in neutral 10% buffered formalin, was cut into 2‐mm‐thick section and paraffin‐embedded. Haematoxylin and eosin‐stained sections were examined. Immunohistochemical staining was performed on the Ventana Benchmark autostainer (Ventana Medical Systems, Tucson, AZ, USA) on 2‐µm‐thick formalin‐fixed paraffin‐embedded sections. Stains for CD19, CD20, CD79a, PAX5, CD10, bcl‐6, bcl‐2, MUM1, CD38, CD138, IgM, IgG, kappa light chain, lambda light chain, CD21, CD2, CD3, CD5, CD30, ALK, granzyme B, ZAP70 and Ki67 were used, according to the manufacturer’s instructions.

HHV8 was detected with an antibody against latent nuclear antigen (also called ORF73), and the presence of EBV was studied with both immunohistochemistry [latent membrane protein (LMP) 1, LMP2, Epstein–Barr nuclear antigen (EBNA) 1), EBNA2, and BZLF‐1] and EBV‐encoded small RNA (EBER) *in‐situ* hybridisation (ISH), performed on the Ventana Benchmark autostainer (Ventana Medical Systems).

The antibody clones, dilutions and antigen retrieval protocols are shown in Table S1. *IGH*–*VDJ* rearrangements were studied in all of the specimens according to the BIOMED‐2 protocol.^26^ In particular, to determine whether the atypical cell population occurring within the germinal centres and the sinuses carried the same rearrangement, laser capture microdissection was performed.^27^, ^28^ The obtained *FR1*‐*JH* polymerase chain reaction (PCR) products of all the samples were directly sequenced and compared with the germline sequence by application of the GenBank database (http://www.imgt.org/IMGT_vquest/vquest).

## Results

### Clinical History

#### Case 1

Multiple abdominal lymph nodes were detected in a 75‐year‐old male during the follow‐up for a low‐grade papillary urothelial carcinoma diagnosed 3 years before. Clinical conditions, laboratory values and haematological values were unremarkable. Serum HIV testing gave negative results, whereas testing for EBV DNA and HHV8 DNA gave positive results (respectively, copy numbers of 327 and 1819 in the blood). An incisional biopsy from an external iliac lymph node was performed, and the diagnosis of HHV8/EBV‐positive EC‐PEL was made, with Castleman’s disease features in the remaining lymph node. No serous effusions were detected. Chemotherapy with rituximab, cyclophosphamide, hydroxydaunorubicin, Oncovin and prednisolone resulted in a complete response after six cycles.

Eighteen months after the first diagnosis, the patient developed pleural effusion; on cytological examination, HHV8/EBV‐positive PEL was diagnosed. No additional therapy was performed; 8 months after the PEL diagnosis, the patient is alive with no evidence of recurrence.

#### Case 2

A 49‐year‐old HIV‐positive African woman presented with enlargement of the bilateral inguinal and para‐aortic lymph nodes. The CD4 count was 439, and the viral load was undetectable. An inguinal lymph node biopsy was performed, and a diagnosis of GLPD and/or early EC‐PEL associated with Castleman’s disease features in the same lymph node was made. No body cavity effusions on computed tomography scan or extranodal involvement were seen. There was no bone marrow involvement. The patient was treated with cyclophosphamide, doxorubicin, and VP16, and underwent complete remission. She is still well after 12 months of follow‐up.

### Histopathological, Immunohistochemical and Molecular Findings

#### Case 1

**Figure 1:**
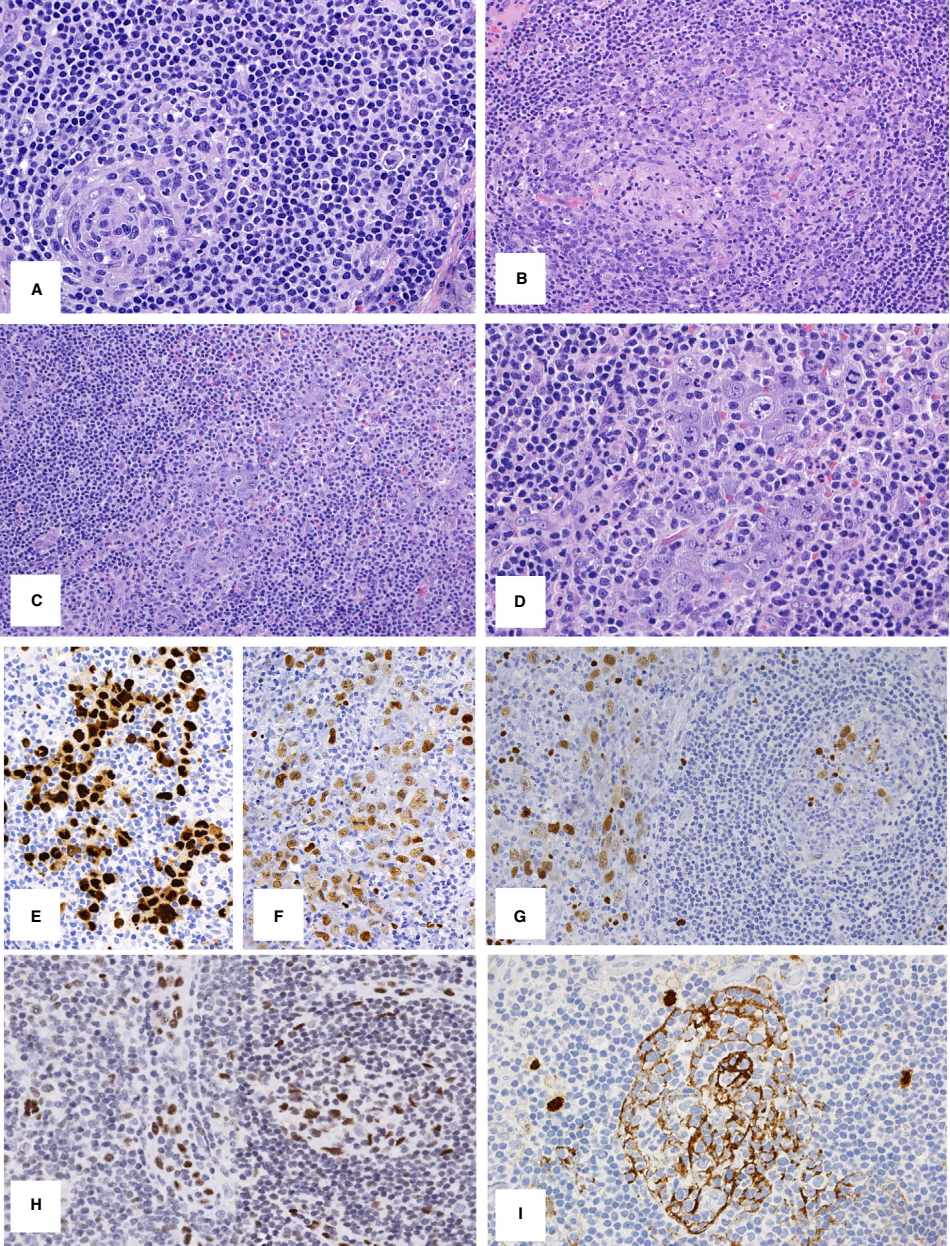
Case 1. **A,** B follicles show regressive germinal centres with hyaline sclerosis, numerous polyclonal plasma cells in the interfollicular areas, and single atypical cells with plasmablastic morphology (OM 20). **B,C,** Focal aggregates of large, atypical cells were identified, with plasmablastic and anaplastic morphology within scattered germinal centres and sinuses (OM 10). **D,** The large, atypical cells showed marked pleomorphic and bizarre nuclei with multiple nucleoli, and abundant eosinophilic cytoplasm (OM 20). **E,F,** The large, atypical cells were positive for human herpesvirus‐8 (HHV8) (**E,** OM 20) and for Epstein–Barr virus‐encoded small RNA (EBER) (**F,** OM 20). **G,H,** In addition, numerous EBV‐positive cells of different sizes were detected in the regressive germinal centres: EBER‐positive (**G,** OM 20) and focally BZLF1‐positive (**H,** OM 20). **I,** Notably, within all germinal centres, the HHV8 stain showed positivity in the form of concentric cytoplasmic processes, probably corresponding to follicular dendritic cells (FDCs) (OM 20).

The lymph node architecture was preserved, with B follicles showing regressive germinal centres with hyaline sclerosis, numerous polyclonal plasma cells in the interfollicular areas, and single atypical cells with plasmablastic morphology (Figure 1A). In scattered germinal centres and in the sinuses, focal aggregates of large, atypical cells were identified, showing marked pleomorphic and bizarre nuclei with multiple nucleoli, abundant eosinophilic cytoplasm, and plasmablastic and anaplastic morphology (Figure 1B–D).

Immunohistochemically, the atypical cells in the sinuses were positive for CD3, but lacked CD2, CD5, CD30, ALK, granzyme B and ZAP70 expression. Staining for CD19, CD20, CD79a, CD138 and PAX5 also gave negative results, whereas the atypical cells expressed MUM1/interferon regulatory factor 4 (IRF4), IgM, and kappa light chain. They were also positive for HHV8 (Figure 1E) and EBV, with heterogeneous expression of EBV latent and lytic gene products [EBER‐positive (Figure 1F), EBNA1‐positive, EBNA2‐negative, LMP1‐negative, LMP2‐negative, and focally BZLF1‐positive]. In addition, numerous EBV‐positive cells of variable size were detected in the regressive germinal centres [EBER‐positive (Figure 1G), EBNA1‐positive, EBNA2‐negative, LMP1‐positive, LMP2‐positive, and focally BZLF1‐positive]. Focal expression of BZLF1 in the sinuses and regressive germinal centres is shown in Figure 1H. Notably, within all germinal centres, the HHV8 stain showed positivity in the form of concentric cytoplasmic processes, probably corresponding to follicular dendritic cells (FDCs), and resembling the patterns of CD21 and CD23 staining (Figure 1I). No nuclear positivity was found in these cells.

Cytological examination of the pleural effusion that developed 18 months later showed pleomorphic anaplastic large cells, which were negative for CD20 and positive for CD38, HHV8, and EBV (Figure S1).

The atypical cells in the sinuses and in the germinal centres were microdissected and analysed with PCR for *JGH* clonality. Whereas the cells occurring in the germinal centres showed an oligoclonal pattern, the intrasinusoidal cells were clearly monoclonal, but a clonal relationship could not be detected. Unfortunately, a comparison of clonality between the nodal lymphoma and PEL was not possible, owing to the low amount and poor quality of DNA in the cytological sample.

#### Case 2

**Figure 2:**
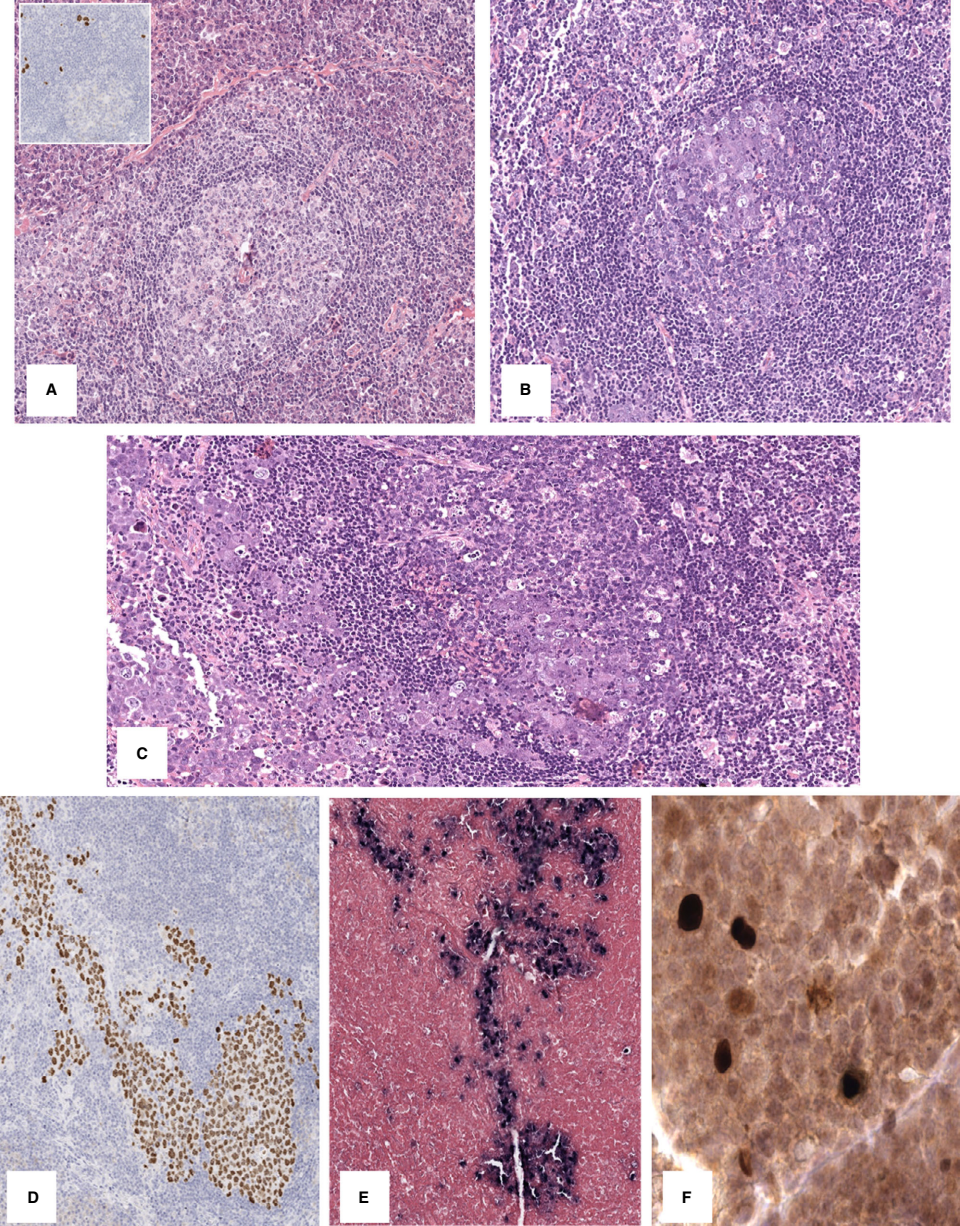
Case 2. **A,** The lymph node architecture was preserved, with large reactive germinal centres and expansion of interfollicular areas with massive infiltrates of polyclonal plasma cells with occasional hyaline vascular features and radially penetrating vessels (OM 10), and single atypical cells with plasmablastic morphology and human herpesvirus‐8 (HHV8) positivity in the mantle zone (inset, OM 20). **B, C,** In other areas, large blastic cells with a considerable degree of plasmablastic differentiation were focally present in scattered germinal centres (**B,** OM 10) and in the sinuses, demonstrating trafficking of the neoplastic cells among them (**C,** OM 10). **D**–**F,** These atypical cells were HHV8‐positive (**D,** OM 5), EBV‐negative (**E,** Epstein–Barr virus‐encoded small RNA *in‐situ* hybridisation, OM 5) and focally BZLF1‐positive (**F,** OM 40).

The lymph node architecture was preserved, with large reactive germinal centres and expansion of interfollicular areas with massive infiltrates of polyclonal plasma cells with occasional hyaline vascular features and radially penetrating vessels (Figure 2A); single atypical cells with plasmablastic morphology and HHV8 positivity were detected in the mantle zone (Figure 2A, inset). In other areas, large blastic cells with vesicular, often eccentrically placed, nuclei, containing one or two prominent nucleoli and amphophilic cytoplasm with a considerable degree of plasmablastic differentiation, were focally present in scattered germinal centres (Figure 2B) and in the sinuses (Figure 2C).

These atypical cells were positive for MUM1/IRF4, kappa light chain, and variably positive for IgM, whereas they did not express CD20, PAX5, CD10, bcl‐6, or CD30. Moreover, they were positive for HHV8 (Figure 2D) and EBV, with variable expression of EBV latent and lytic gene products [positive for EBER‐ISH (Figure 2E), EBNA1‐positive, EBNA2‐negative, focally BZLF1‐positive (Figure 2F), LMP1‐negative, and LMP2‐negative].

PCR analysis for *JH* clonality on the atypical cells occurring in the sinuses and in the germinal centres showed an identical *IGH* rearrangement with somatic hypermutations.

## Discussion

Individual cases of HHV8‐related LPD showing overlapping features have been reported.^9^ Such cases may well represent particular diagnostic challenges, particularly regarding the distinction between MCD with plasmablastic aggregates (previously termed ‘microlymphoma’), GLPD, and EC‐PEL. Here, we report two cases of LPD associated with HHV8 and EBV that further expand the spectrum of HHV8/EBV‐positive lymphoproliferations^29^, ^30^, ^31^, ^32^, ^33^, ^34^, ^35^, ^36^, ^37^, ^38^, ^39^ (Table S2). Thus, HHV8/EBV‐positive LPDs probably represent a broad spectrum of lesions with overlapping features and possible evolution among different entities^29^, ^30^, ^31^, ^32^, ^33^, ^34^, ^35^, ^36^, ^37^, ^38^, ^39^ (Table S3).

Case 1 arose in an immunocompetent patient who initially presented with asymptomatic and isolated abdominal lymphadenopathy. The striking characteristic of this case was the almost exclusive intrasinusoidal involvement of the neoplastic cells and the association with Castleman’s disease features. The intrasinusoidal growth pattern and the strong expression of CD3 simulated an anaplastic large‐cell lymphoma,^23^ but the atypical cells expressed MUM1/IRF4, IgM, and kappa light chain, and were HHV8‐positive and EBV‐positive. On the basis of these findings, the diagnosis of HHV8/EBV‐positive EC‐PEL was favoured. Notably, after 18 months, the patient developed pleural effusion corresponding to PEL. EC‐PEL can precede or follow a classic case of PEL, usually in HIV‐positive patients.^10^ Although we could not demonstrate a clonal relationship between the two lesions, we can hypothesise that the intrasinusoidal lymph node involvement represents an early phase of PEL, rather than two independent HHV8/EBV‐positive neoplasms. In this particular case, an additional interesting feature was represented by the occurrence of numerous EBV‐positive/HHV8‐negative cells in regressive germinal centres, whose significance and relationship with the HHV8/EBV‐positive lymphoma is difficult to define.^40^ However, the intrafollicular distribution and the immunophenotype of EBV‐positive cells, as well as their EBV latency protein expression pattern and lack of overt *IGH* clonality, favour reactivation of EBV infection.

Interestingly, positivity for HHV8 was detected along the FDC cytoplasmic processes. There is experimental evidence that dendritic cells and macrophages can be infected through the DC‐SIGN receptor expressed on their surfaces. However, we were unable to demonstrate a clear nuclear stain giving evidence of HHV8 infecting FDCs.^41^ Our finding may, rather, represent the uptake of HHV8 antigen by FDCs, as reported in some cases of MCD and during HIV infection.^42^, ^43^

Dendritic cells play an important role in HHV8 infection and pathogenesis. However, it is not yet clear how this might alter the immune response associated with HHV8 and how it influences HHV8‐related disease development.^44^, ^45^

In the second case, we found the entire spectrum of HHV8‐related disorders (MCD, GLPD, and PEL) coexisting in the same lymph node, underlining the variability, the overlap and the possible evolution among these entities. Cases with clinicopathological features of MCD enriched for plasmablastic aggregates and GLPD have been reported, suggesting an overlap with MCD and GLPD in a subset of cases.^25^, ^34^ Although GLPD progressing to lymphoma has been reported in HIV‐positive and HIV‐negative patients,^29^ definitive association and evolution from MCD to GLPD or EC‐PEL is still not recognised.^46^ In our case we were able to demonstrate a clonal relationship between the atypical large cells in the germinal centres and in the sinuses from the same lymph node.

HHV8/EBV coinfection may be a key factor in explaining the overlap and evolution among HHV8/EBV‐positive LPDs.^4^ HHV8 would infect naive B cells, establishing a reservoir of polyclonal B cells showing features of mature B cells.^15^, ^47^, ^48^, ^49^ HHV8/EBV coinfection may thus influence the occurrence of an LPD, with subsequent clonal evolution towards an overt lymphomatous proliferation with a post‐germinal centre phenotype, complex karyotype and somatic hypermutations under the influence of EBV reactivation.^4^, ^16^, ^50^, ^51^

EBV has different latency programmes, and both latent and lytic genes may have an oncogenic effect. Interestingly, in our case we observed a heterogeneous EBV latency programme, with expression also of the lytic gene *BZLF1*. This non‐canonical latency programme has been observed in other lymphoma subtypes.^28^, ^52^, ^53^ When the EBV oncogenic products are expressed at a particular step of B‐cell differentiation and in a special microenvironment, fatal malignant events may occur (Figure S2). Our findings are in line with the concept that EBV reactivation and expression of BZLF1 may influence clonal evolution in HHV8‐related LPD, and further confirm the experimental evidence that lytic EBV replication augments HHV8‐related tumorigenesis, underlying the interaction between these two oncogenic viruses also in humans.^17^

Cases of MCD associated with EBV have been reported,^54^ and we have also recently observed a case of MCD with HHV8‐positive cells in the mantle areas, and EBV reactivation with expression of BZLF1 in germinal centre cells (Figure S3). Such cases might warrant stricter follow‐up for possible transformation in LPDs associated with HHV8 and EBV.

In summary, our cases varied from the usual morphology and clinical scenario of HHV8‐related LPDs, potentially leading to misdiagnosis. The present cases broaden the spectrum of HHV8/EBV lymphoproliferation and underline the grey zone among HHV8‐related LPDs.

## Conflicts of interest

The authors declare that they have no conflict of interest.

## Author contributions

GA, SA, UM: provided tumour samples and clinical data; ML, AT, SE: performed immuhistochemestry and cytogenetic analysis; GM, FM, MV, BC, UM, FF, MRL, LL, LS: analysed and interpreted the data; GM, FM, FF, MRL, LL, LS: designed and coordinate the study; GM, FM, FF, LL, LS: interpreted the data and wrote the manuscript.

## Supporting information


**Figure S1**. Case 1, pleural effusion.

**Figure S2**. Cartoon illustrating progression of human herpesvirus 8 (HHV)‐positive multicentric Castleman disease to a broad spectrum of lesions with overlapping features.

**Figure S3**. A case of MCD associated with EBV.

**Table S1**. Antibodies used.

**Table S2**. The differential diagnosis of HHV8‐positive lymphoproliferative disorders.

**Table S3**. Summary of the literature review.
