# Supplementary figures and images for: Epstein–Barr virus reactivation influences clonal evolution in human herpesvirus‐8‐related lymphoproliferative disorders
Source: Histopathology. 2021 Oct 4;79(6):1099–107. doi: 10.1111/his.14551 (PMC9293042; doi:10.1111/his.14551)

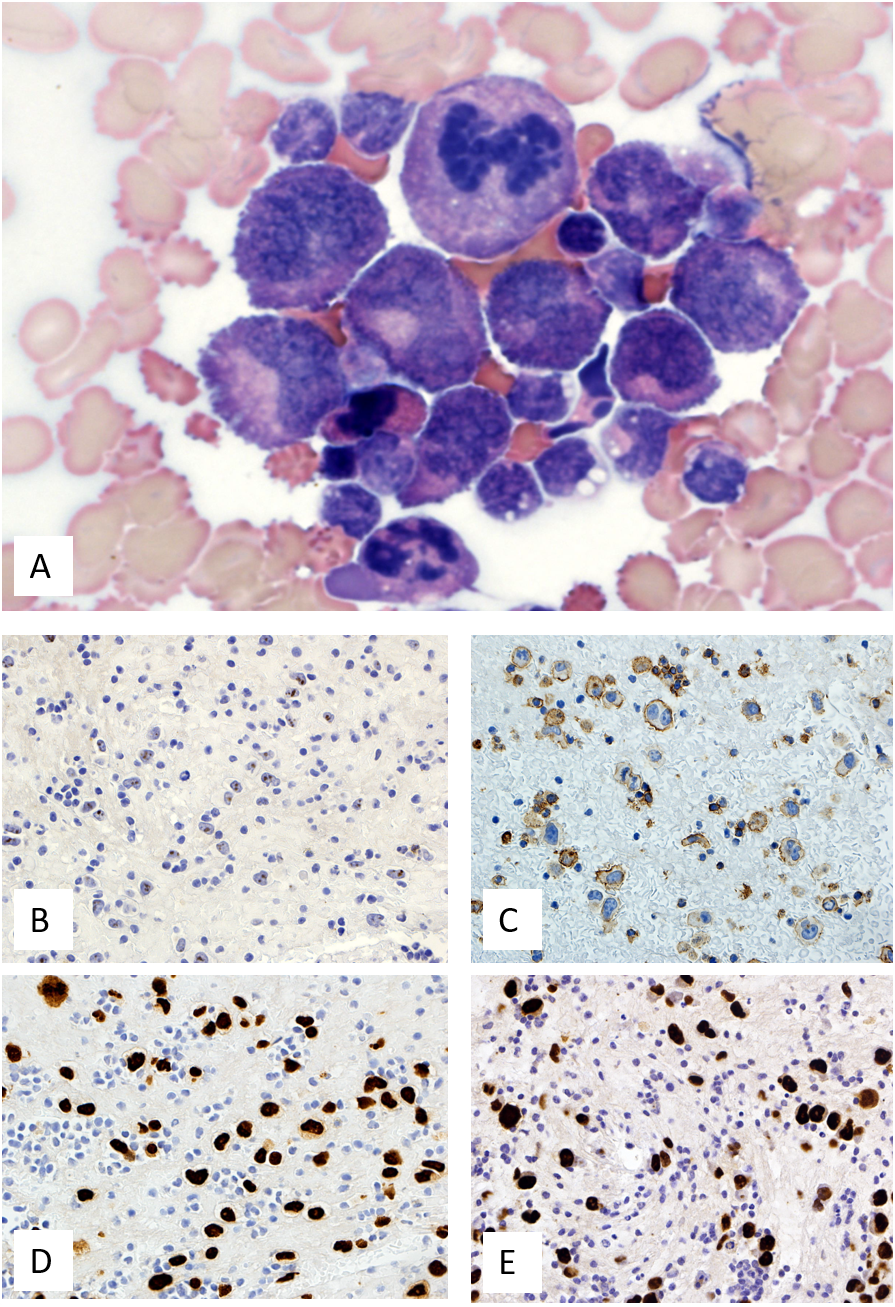

Supplement: Supplementary file 1 — Figure S1. Case 1, pleural effusion. [file HIS-79-1099-s001.tif]

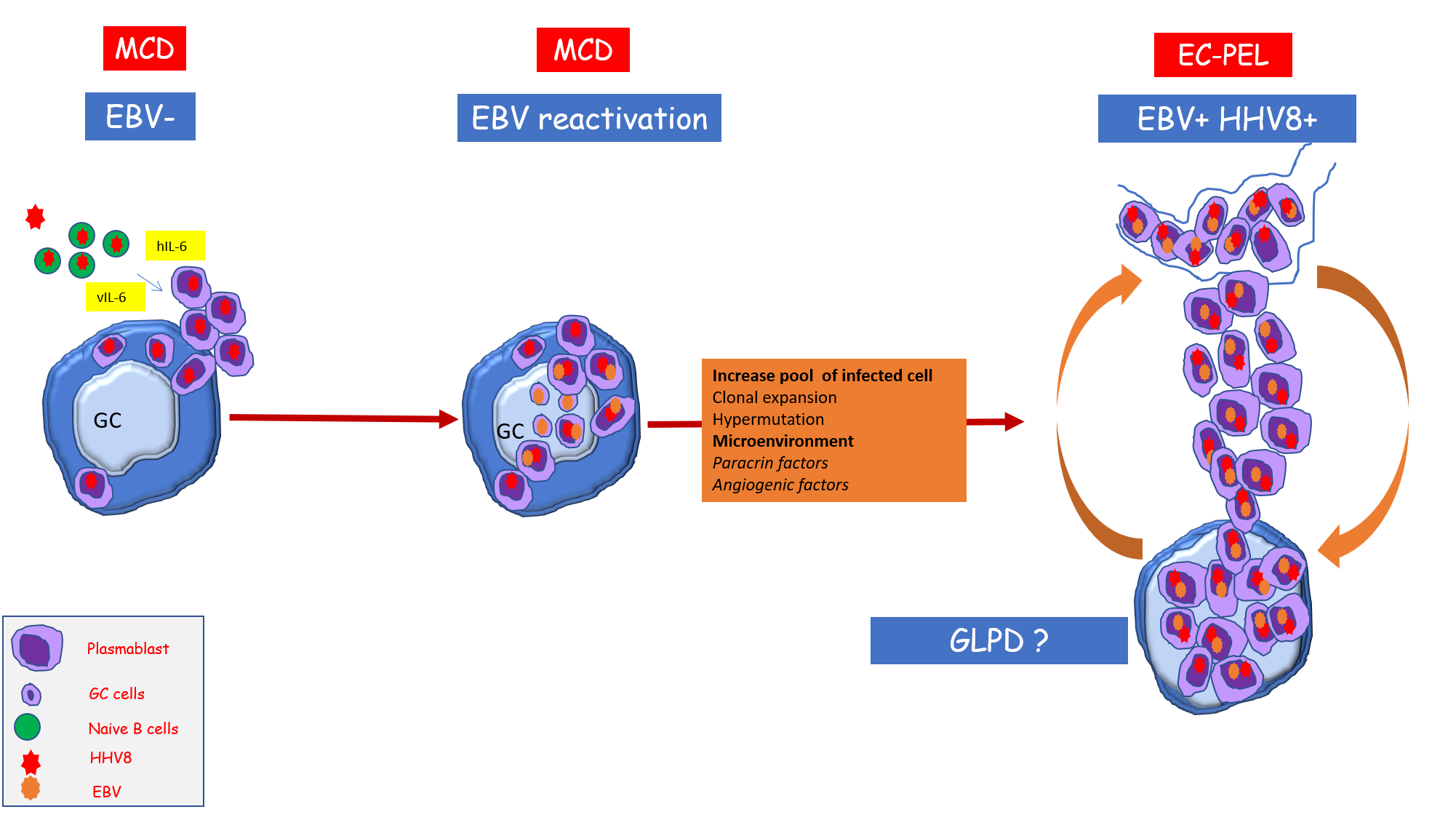

Supplement: Supplementary file 2 — Figure S2. Cartoon illustrating progression of human herpesvirus 8 (HHV)‐positive multicentric Castleman disease to a broad spectrum of lesions with overlapping features. [file HIS-79-1099-s005.tif]

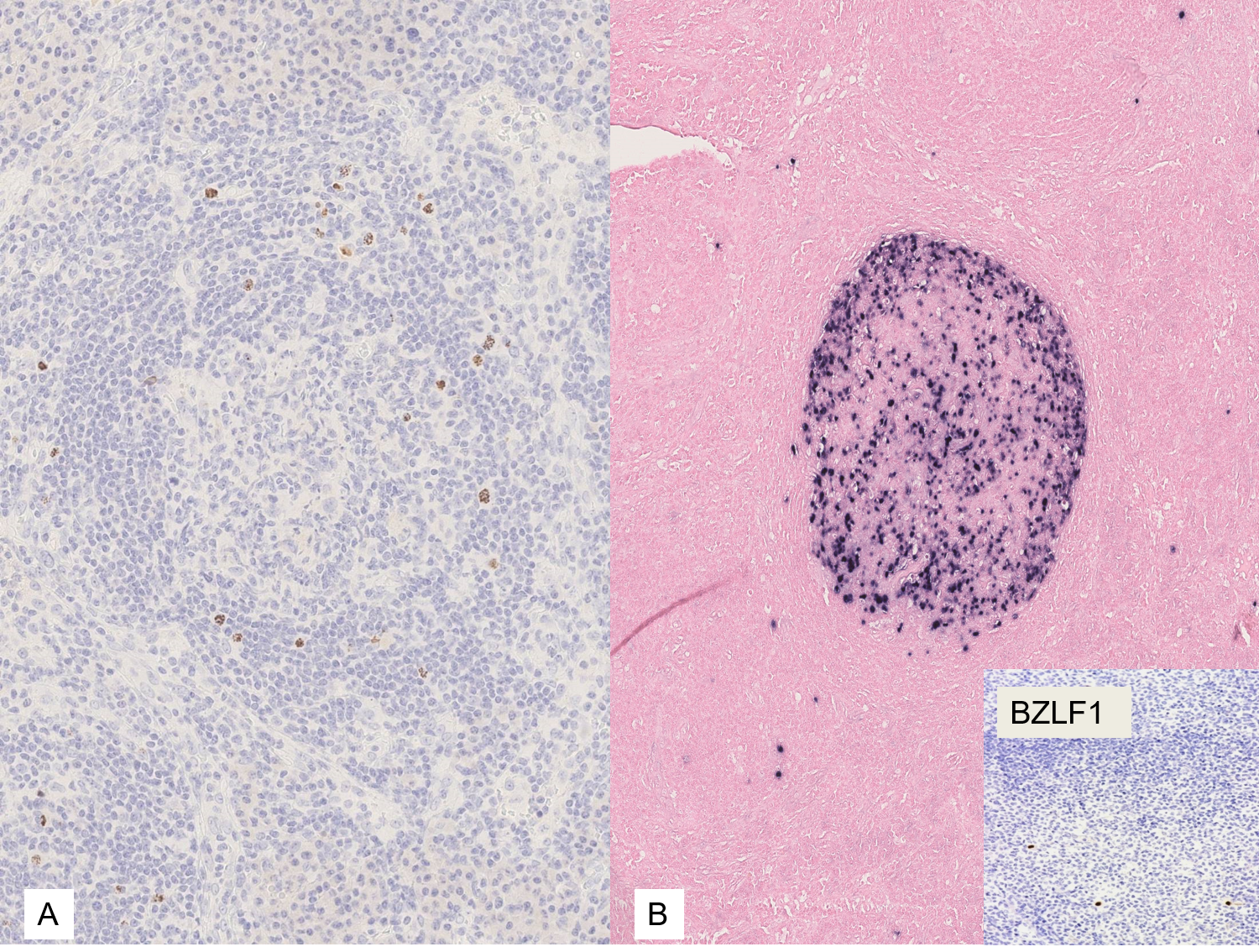

Supplement: Supplementary file 3 — Figure S3. A case of MCD associated with EBV. [file HIS-79-1099-s004.tif]
