# Supplementary material for: Epstein–Barr virus reactivation influences clonal evolution in human herpesvirus‐8‐related lymphoproliferative disorders
Source: Histopathology. 2021 Oct 4;79(6):1099–107. doi: 10.1111/his.14551 (PMC9293042; doi:10.1111/his.14551)
Supplement: Supplementary file 4 — Table S1. Antibodies used. [file HIS-79-1099-s006.docx]

| **ANTIBODY** | **COMPANY** | **CLONE** | **DILUITION** | **INCUBATION** |
| --- | --- | --- | --- | --- |
| ALK | VENTANA | ALK 01 | 1:100 | 28 MINUTES |
| BCL-2 | VENTANA | SP66 | 1:20 | 30 MINUTES |
| BCL-6 | CELL MARQUE | GII91E/A8 | 1:50 | 28 MINUTES |
| CD 10 | VENTANA | SP67 | READY TO USE | 30 MINUTES |
| CD 138 | VENTANA | B-A38 | READY TO USE | 40 MINUTES |
| CD 19 | CELL MARQUE | MRQ-36 | 1:50 | 28 MINUTES |
| CD 2 | CELL MARQUE | MRQ-11 | 1:100 | 12 MINUTES |
| CD 20 | VENTANA | L-26 | READY TO USE | 40 MINUTES |
| CD 21 | VENTANA | 2G9 | 1:20 | 20 MINUTES |
| CD 30 | CELL MARQUE | BER-H2 | 1:50 | 40 MINUTES |
| CD 38 | CELL MARQUE | SP149 | READY TO USE | 28 MINUTES |
| CD 5 | VENTANA | SP19 | READY TO USE | 12 MINUTES |
| CD 79a | VENTANA | SP18 | READY TO USE | 20 MINUTES |
| GRANZYME B | ABCAM | AB4059 | 1:150 | 28 MINUTES |
| HHV8 | VENTANA | 13B10 | READY TO USE | 40 MINUTES |
| IGM | CELL MARQUE | 2654 | READY TO USE | 12 MINUTES |
| IGG | CELL MARQUE | 2653 | READY TO USE | 12 MINUTES |
| KAPPA | CELL MARQUE | L1C1 | 1:20 | 28 MINUTES |
| KI-67 | VENTANA | 30 9 | READY TO USE | 40 MINUTES |
| LAMBDA | CELL MARQUE | LAMB14 | 1:50 | 28 MINUTES |
| LMP1 | CELL MARQUE | CS1-4 | 1:20 | 40 MINUTES |
| LMP2 | SANTA CRUZ | SC-373971 | 1:50 | 40 MINUTES |
| EBNA 1 | SANTA CRUZ | SC-81581 | 1:250 | 40 MINUTES |
| EBNA2 | ABCAM | AB 90543 | 1:50 | 40 MINUTES |
| BZLF-1 | SANTA CRUZ | SC-53904 | 1:100 | 28 MINUTES |
| MUM 1 | BIOSYSTEM | MUM1p | 1:100 | 12 MINUTES |
| PAX5 | VENTANA | SP34 | 1:20 | 40 MINUTES |
| ZAP-70 | CELL MARQUE | 2F3.2 | 1:100 | 40 MINUTES |
|  |  |  |  |  |

Antibodies Table
