# Supplementary material for: Epstein–Barr virus reactivation influences clonal evolution in human herpesvirus‐8‐related lymphoproliferative disorders
Source: Histopathology. 2021 Oct 4;79(6):1099–107. doi: 10.1111/his.14551 (PMC9293042; doi:10.1111/his.14551)
Supplement: Supplementary file 5 — Table S2. The differential diagnosis of HHV8‐positive lymphoproliferative disorders. [file HIS-79-1099-s002.docx]

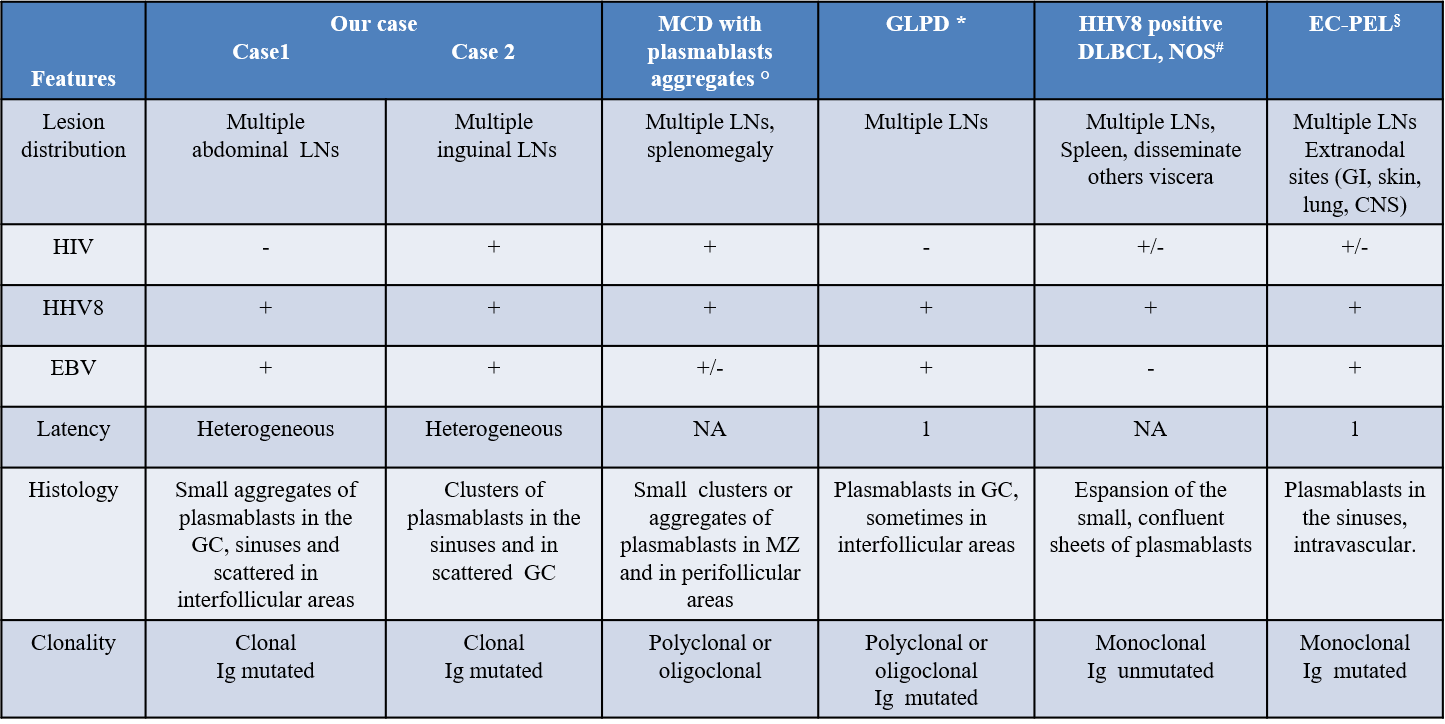


° ref 29; * ref 35; # ref 21; §ref. 23

DLBCL: diffuse large B cell lymphoma; EC-PEL: extracavitary primary effusion lymphoma; GC: germinal center;

GLPD: germinotropic lymphoproliferative disorders; Ig: immunoglobulins; LN: lymph node; MCD: multicentric

Castleman disease; MZ: mantle zone; NA: not applicable

The differential diagnosis of HHV8-positive lymphoproliferative disorders
