# Supplementary material for: Epstein–Barr virus reactivation influences clonal evolution in human herpesvirus‐8‐related lymphoproliferative disorders
Source: Histopathology. 2021 Oct 4;79(6):1099–107. doi: 10.1111/his.14551 (PMC9293042; doi:10.1111/his.14551)
Supplement: Supplementary file 6 — Table S3. Summary of the literature review. [file HIS-79-1099-s003.docx]

Summary of the literature review

| Dupin N, Diss TL, Kellam P , et al. (2000) (ref 48) | HHV-8 is associated with a plasmablastic variant of Castleman disease that is linked to HHV-8-positive plasmablastic lymphoma. | Plasmablastic lymphoma associated with MCD is a new disease entity associated with HHV-8 infection. |
| --- | --- | --- |
| Du M Q, Liu H, Diss T C, et al. (2001) (ref 47) | Kaposi sarcoma-associated herpesvirus infects monotypic (IgM lambda) but polyclonal naive B cells in Castleman disease and associated lymphoproliferative disorders. | Activation of the IL-6 receptor signaling pathway may play a role in differentiation of KSHV-infected naive B cells into plasmablasts and development of lymphoproliferative lesions. |
| Chadburn A, Hyjek E, Mathew S, et al. (2004 ) (ref 8) | KSHV-positive solid lymphomas represent an extra-cavitary variant of primary effusion lymphoma | These findings strongly suggest that these decidedly rare KSHV-positive solid lymphomas belong to the spectrum of PEL. Therefore, we propose that the KSHV-positive solid lymphomas be designated extra-cavitary PELs. |
| Seliem RM, Griffith RC, Harris NL, et al. (2007)  (ref 22) | HHV-8+, EBV+ multicentric plasmablastic microlymphoma in an HIV+ Man: the spectrum of HHV-8+ lymphoproliferative disorders expands. *Am J SurgPathol*.;31(9):1439-1445. | This unique case has clinical features compatible with a MCD-associated plasmablastic lymphoproliferative disorder, with pathologic features intermediate between HHV-8+ plasmablastic microlymphoma, and HHV-8+ germinotropic lymphoproliferative disorder. |
| Chadburn A, Hyjek E M, Tam W, et al. (2008) (ref 49) | Immunophenotypic analysis of the Kaposi sarcoma herpesvirus (KSHV; HHV-8)-infected B cells in HIV+ multicentric Castleman disease (MCD) | Although both PEL and MCD originate from KSHV-infected pre-terminally differentiated B cells, these findings, with previously reported genetic studies, indicate HIV+ MCD may arise from extrafollicular B cells, whereas PELs may originate from cells that have traversed the germinal centre. |
| Ferry JA, Sohani AR, Longtine JA, et al (2009)  (ref 30) | HHV8-positive, EBV-positive Hodgkin lymphoma-like large B-cell lymphoma and HHV8-positive intravascular large B-cell lymphoma | On the basis of the histologic features in these two cases, the presence of HHV8 was unexpected. These cases expand the spectrum of lymphoproliferative disorders that can be associated with HHV8. |
| Peker D, Alkan S, Zhang L, *et al* (2013).  (ref 31) | HIV-associated plasmablastic multicentric Castleman disease with microlymphoma coinfected with HHV8 and EBV. | This case has clinical features compatible with a rare type of HHV8+ and EBV + MCD-associated plasmablastic lymphoproliferative disorder with a germinotropic pattern showing possible MYC gene deregulation. |
| Courville EL, Sohani AR, Hasserjian RP, et al. 2014 Dec;142(6):816-29. (ref 23) | Diverse Clinicopathologic Features in Human Herpesvirus 8–Associated Lymphomas Lead to Diagnostic Problems. | HHV8-associated lymphomas can be clinically and pathologically heterogeneous, with features that may lead to misdiagnosis as other types of lymphoma. |
| Papoudou-Bai A, Hatzimichael E, Kyriazopoulou L, et al. (2015) (ref 24) | Rare variants in the spectrum of human herpesvirus 8/Epstein-Barr virus–copositive lymphoproliferations. | The present cases broaden the spectrum of HHV-EBV--copositive lymphoproliferations. |
| Gonzalez-Farre B, Martinez D, Lopez-Guerra M, et al*. (*2017) (ref 29) | HHV8-related lymphoid proliferations: a broad spectrum of lesions from reactive lymphoid hyperplasia to overt lymphoma. | These findings expand the clinical and pathological spectrum of HHV8-related lymphoid proliferations, which is broader than current recognized. |
| Lee YM, Kim JM, Kim SY.. *J* (2017) (ref 32) | Human Herpes Virus 8/Epstein-Barr Virus-Copositive, Plasmablastic Microlymphoma Arising in Multicentric Castleman's Disease of an Immunocompetent Patient | We describe a plasmablastic microlymphoma arising in MCD showing HHV8/EBV co-positivity especially in HIV-seronegative patient. |
| Bacha D, Chelly B, Kilani H, et al. *(2017) (ref 33)* | HHV8/EBV Coinfection Lymphoproliferative Disorder: Rare Entity with a Favorable Outcome. | Through the review of the nine previously reported cases in literature, we discuss the clinical and pathologic features and the differential diagnosis of HHV8/EBV GLD. |
| Wang W, Kanagal-Shamanna R, Medeiros LJ. (2018)(ref 36) | Lymphoproliferative disorders with concurrent HHV8 and EBV infection: beyond primary effusion lymphoma and germinotropic lymphoproliferative disorder | The description of these two cases suggests that the category of HHV8+ EBV+ LPDs is a spectrum, which not only includes PEL and germinotropic LPD, but also other cases that have overlapping but distinctive features. |
| Zanelli M, Fraternali Orcioni G, Zizzo M, et al (2019) (ref 35) | HHV-8- and EBV-positive germinotropic lymphoproliferative disorder. | Interestingly our case showed MCD-like features as atrophic, hyalinized GC with vascular proliferation suggesting a possible overlap between GLD and MCD |
| Guerrero C, Jain T, Kelemen K. (2019) (ref 44) | HHV-8-associated lymphoprolipherative disorders and pathogenesis in an HIV-Positive Patient | Recently discovered variants of HHV-8-associated LPDs indicate that this group represents a diverse spectrum of disorders, whose classification may require further refinement beyond the currently recognized entities. |
| Vega, F, Miranda RN, Medeiros LJ. (2020) (ref 21) | KSHV/HHV8-positive large B-cell lymphomas and associated diseases: a heterogeneous group of lymphoproliferative processes with significant clinicopathological overlap. | It is important to have a better understanding of the biology of these lesions and to refine diagnostic criteria of these lesions. |
| Martinez-Ciarpaglini C, Valkov A, Hurtado M, et al. (2020) (ref 25) | Intrasinusoidal HHV8-EBV–Positive Large B-Cell Lymphoma With Features of Germinotropic Lymphoproliferative Disorder | Our findings contribute to the expansion of the morphological spectrum of HHV8-associated lymphoproliferative lesions and aids in the characterization of the very infrequent GLPD entity. |
| Sanchez S, Veloza L, Luojun W, et al. (2020) (ref 37) | HHV8-positive, EBV-positive Hodgkin lymphoma-like large B cell lymphoma: expanding the spectrum of HHV8 and EBV-associated lymphoproliferative disorders | This case presents diagnostic challenges due to the presence of particular features not clearly related to current HHV8-associated entities, and also suggests the possibility for disease progression in the spectrum of HHV8- and EBV-associated lymphoproliferative disorders. |
| Nakaya Y, Ishii N, Kasamatsu Y, et al. (2020) (ref 34) | Human herpesvirus 8-positive multicentric Castleman disease with germinotropic plasmablastic aggregates: Overlapping spectrum of human herpesvirus 8-associated lymphoproliferative disorder. | The pathogenesis of each HHV8-associated LPD differs in cellular origin, host immune status, cytoplasmic immunoglobulin expression, clonality pattern and EBV infection; however, these factors sometimes overlap and induce extended clinical and pathologic presentations. |
